# Applying Ensemble Ecological Niche Modeling to Identify High Risk Areas for Scorpions' Sting

**DOI:** 10.1002/ece3.71713

**Published:** 2025-07-04

**Authors:** Anooshe Kafash, Ahmad Ali Hanafi Bojd, Anna Pintor, Marc Grünig, Masoud Yousefi, Gholamreza Hassanpour

**Affiliations:** ^1^ Zoonosis Research Center Tehran University of Medical Sciences Tehran Iran; ^2^ Department of Medical Entomology and Vector Control, School of Public Health Tehran University of Medical Sciences Tehran Iran; ^3^ Australian Institute of Tropical Health and Medicine James Cook University Cairns Australia; ^4^ Swiss Tropical and Public Health Institute (Swiss TPH) Allschwil Switzerland; ^5^ Faculty of Governance University of Tehran Tehran Iran; ^6^ Center for Research of Endemic Parasites of Iran (CREPI) Tehran University of Medical Sciences Tehran Iran

**Keywords:** Iran, niche models, scorpions, sting risk

## Abstract

Scorpion stings are a global health problem, potentially impacting thousands, especially across northern Africa and the Middle East. However, scorpions are comparatively understudied compared to other venomous animals, and little is known about their distributions and associated spatial patterns of sting risk. Timely access to medical treatment after scorpion stings is crucial to positive medical outcomes, but it remains poorly understood how accessible health care is for populations at risk. Thus, mapping hotspots of sting risk and identifying vulnerable populations can greatly enhance mitigation strategies. In this study, we used ecological niche models to map the distribution of eight of the most dangerous scorpion species to quantify sting risk across Iran. We identified the areas where people are most vulnerable to scorpion stings based on exposure to scorpions and limited accessibility to healthcare centers. We identified sting risk areas for each species across their distribution ranges. *Androctonus crassicauda* has the widest distribution range. On the contrary, *Orthochirus iranus* has the smallest distribution range. Regions of greatest vulnerability are located in the central parts of Iran, as well as the south and southwest of the country. Our results provide valuable information for decision makers by supporting targeted awareness‐raising programs and antivenom distribution among the most vulnerable populations.

## Graphical abstract

In this study, we used ecological niche models to map the distribution of eight of the most dangerous scorpion species to quantify sting risk across Iran. We identified the areas where people are most vulnerable to scorpion stings based on exposure to scorpions and limited accessibility to healthcare centers. Our results provide valuable information for decision makers by supporting targeted awareness‐raising programs and antivenom distribution among the most vulnerable populations.

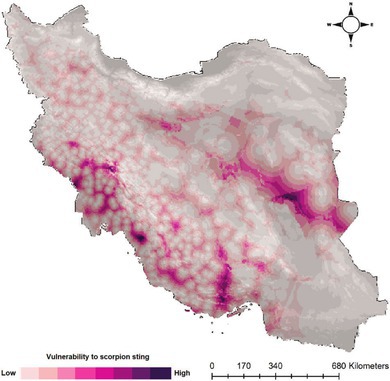

## 1 Introduction

Scorpions (Arachnida: Scorpiones Koch, 1837), dating back 433–438 million years (Jason and Paul 2013; Waddington et al. 2015), are a diverse arachnid group with around 2885 species globally (https://www.ntnu.no/ub/scorpion‐files/). Originating from the Paleozoic era, they have evolved to inhabit almost every landmass, from deserts to forests, except Antarctica (Dunlop 2010; Howard et al. 2019). Scorpions thrive in various climates, adapting to extreme temperatures and environments, but most species prefer warm climates. One particular characteristic of scorpions is their ability to produce venom (Arbuckle 2017; Senji Laxme et al. 2019). Scorpion stings, while rarely fatal, pose significant health challenges (Isbister and Bawaskar 2014). In case of a scorpion sting, it is crucial to receive medical treatment in a short time. While this is possible in populated areas with good medical infrastructure, rural areas often have limited access to medical care (Chippaux and Goyffon 2008). Importantly, scorpion stings are not currently listed as a neglected tropical disease, as opposed to snakebite, despite being a substantial and often underappreciated health concern in many countries. Consequently, resources for research on the topic are sparse, and our understanding of the full extent of the issue is limited. Unfortunately, limited understanding and case number recording can lead to a lack of recognition as a significant and widespread medical condition.

To mitigate scorpion sting impact, education on the risks and prevention is vital. Strategies include raising awareness about avoiding scorpion habitat, using protective clothing, and implementing effective antivenom treatments. But to perform awareness‐raising programs, areas where scorpion sting risk (SSR) is high need to be targeted (Kafash et al. 2023). To identify vulnerable populations, the accessibility to health facilities is an important factor in addition to SSR levels. Healthcare centers are not equally distributed and are mostly located in populated areas where the risk is quite low. High SSR areas are strongly linked to the occurrence and abundance of venomous scorpion species, and SSR levels can be estimated using Ecological Niche Models (ENMs) (Pintor et al. 2021; Yousefi, Yousefkhani, et al. 2023). However, to calibrate these models, reliable occurrence records are paramount. Such models are often the most viable option to assess the overlap of human populations with venomous animals and, consequently, the risk of envenomation (Kafash et al. 2023; Rafinejad et al. 2020), because collecting data on actual envenomation events is difficult without country‐wide obligatory reporting systems in place.

Ecological niche models (Guisan et al. 2017) are increasingly being used in health geography (Archer et al. 2023; Barker and MacIsaac 2022; Bozorg‐Omid et al. 2023; Liu et al. 2022; Valderrama et al. 2021; Yousefi, Yousefkhani, et al. 2023). These models use occurrence data of venomous species, vectors, and reservoirs along with environmental data like climate, topography, vegetation, and soil to predict suitable areas of these organisms that cause significant threats to public health (Bozorg‐Omid et al. 2023; Kafash et al. 2023; Liu et al. 2022; Valderrama et al. 2021). Previously, these models have been successfully used in identifying potential areas for past and current distributions of scorpions, determining high SSR areas, human settlements at risk of scorpion stings, and predicting the impacts of climate change on scorpions and SSR patterns (Graham et al. 2013; Hanafi‐Bojd et al. 2020; Kafash et al. 2023; Rafinejad et al. 2020; Ureta et al. 2020).

Iran is a biodiversity‐rich country hosting a high diversity of species, including venomous species like scorpions (Barahoei et al. 2020; Yousefi, Yousefkhani, et al. 2023). So far, more than 83 scorpions have been identified in Iran (Barahoei et al. 2020). While some scorpion species are known to be of medical importance, little is known about the SSR patterns in the country because some species have restricted ranges or remote distributions in areas without human settlements (Barahoei et al. 2020). Considering that scorpion sting is a major health challenge in Iran (Dehghani and Arani 2015; Dehghani et al. 2009; Dehghani and Fathi 2012; Dehghani and Kassiri 2017; Firoozfar et al. 2019; Kafash et al. 2023; Nejati et al. 2014; Rafinejad et al. 2020; Shahsavarinia et al. 2017), the aims of this study are: (i) to identify the most dangerous species in Iran from published literature; (ii) to model the habitat suitability of these species using state‐of‐the‐art ENM modeling techniques; and (iii) identify high SSR areas and vulnerable populations by jointly considering exposure to scorpions and distance to health facilities.

## 2 Materials and Methods

### 2.1 Study Area

Located at the crossroad of the three biogeographic realms, Palearctic, Afrotropic, and Indomalaya, Iran is a large and biodiverse country in southwest Asia (Yousefi, Yousefkhani, et al. 2023). The country is known for its diverse climatic, topographic, and edaphic conditions (Yousefi, Yousefkhani, et al. 2023). Altitude range changes from −26 to 5770 m, and the main part of the country has a height of more than 1200 m. Annual mean precipitation varies from below 50 mm to above 2000 mm, and annual mean temperature ranges from 7.1°C to 27.6°C across the country (Kafash et al. 2020; Yousefi, Mahmoudi, et al. 2023). Iran's population is about 87.92 million (2021), and 45% live in rural areas. Southern parts of the country have a subtropical climate, making the country a suitable area for thermophilous species, including scorpions (Barahoei et al. 2020).

### 2.2 Medically Important Scorpions List and Distribution Data

**FIGURE 1:**
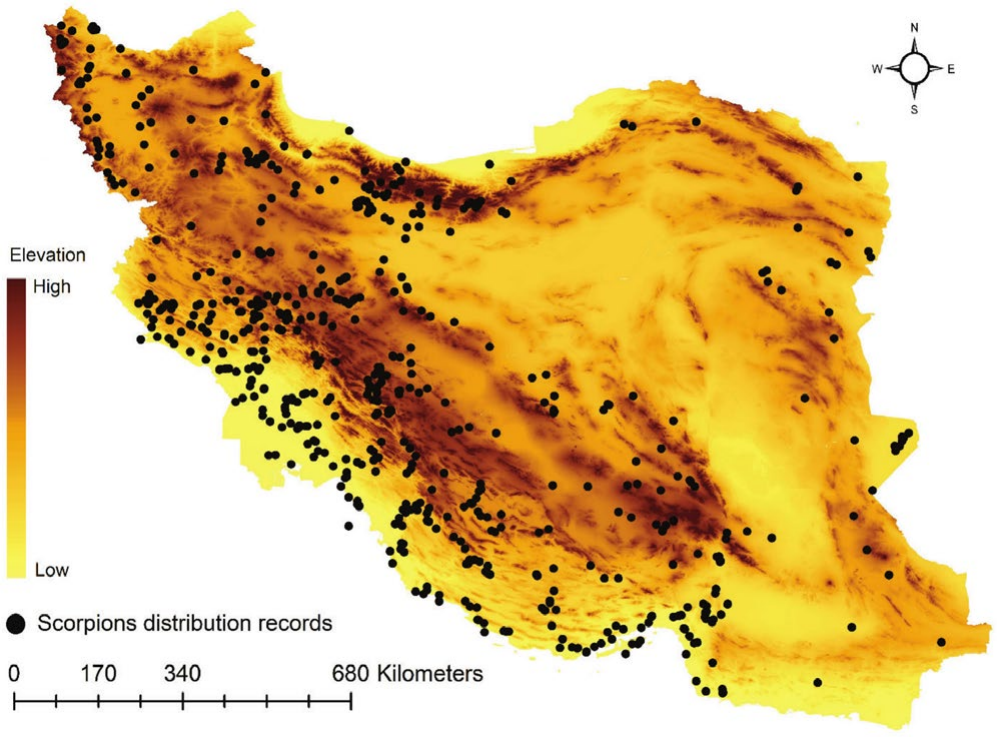
Study area and distribution records of medically important scorpions, shown on the digital elevation model.

We did a literature review to list medically important scorpions in Iran. Our literature review revealed 18 species recognized for their medical importance, with reports of concern from at least some local hospitals (Bavani et al. 2017; Dehghani et al. 2009; Mohammadi Bavani et al. 2020). We were unable to collect enough distribution records for all 18 species, and some uncertainty is associated with the identification of all these 18 species as medically important scorpions. Thus, we continued our analysis with the eight scorpions (Table 1) for which we were able to collect enough reliable distribution records (Figure 1). The scorpions' distribution records were obtained from the Delta database (Barahoei et al. 2020) which is the most comprehensive and up‐to‐date database of scorpion species in Iran (Table S1). The distance between the points of presence was reduced to at least 1 km to match the spatial resolution of environmental layers and to minimize spatial autocorrelation.

**TABLE 1:** List of eight medically important scorpions modeled in this study.

Family	Genus	Species	Number of distribution records
Buthidae	*Androctonus*	*Androctonus crassicauda*	148
	*Buthacus*	*Buthacus macrocentrus*	43
	*Mesobuthus*	*Mesobuthus caucasicus*	30
		*Mesobuthus eupeus*	271
		*Mesobuthus phillipsii*	121
	*Orthochirus*	*Orthochirus iranus*	43
Hemiscorpiidae	*Hemiscorpius*	*Hemiscorpius acanthocercus*	15
		*Hemiscorpius lepturus*	97

### 2.3 Environmental Data

In order to model ecological niches of medically important scorpions in Iran we used CHELSA bioclimatic variables at 30‐s (~1 km) spatial resolution (Karger et al. 2017). As predictor variables for niche modeling, we selected five variables including growing degree days heat sum above 10°C (GDD), temperature seasonality (Bio4), annual precipitation (Bio12) and precipitation seasonality (Bio15) and topographic heterogeneity. These variables were selected because of their known biological relevance for arthropods and their minimal multicollinearity based on the variance inflation factor (VIF) (VIF < 10). Topographic heterogeneity was calculated from the Shuttle Radar Topography Mission (SRTM) digital elevation model (Jarvis et al. 2008) with a 30 arcsec resolution using the raster package of R (Hijmans 2021).

### 2.4 Ecological Niche Modeling

To model the habitat suitability of the study species, we used an ensemble approach with five different modeling algorithms. We considered generalized linear models (GLMs) using the *base* R package (Team 2021), generalized additive models (GAMs) using the *gam* R package version 1.20.1 (Hastie 2022), generalized boosted models (GBMs) using the *gbm* R‐package version 2.1.8 (Greenwell et al. 2020), random forests (RFs) using the *randomForest* R package version 2.1.8 (Liaw and Wiener 2002) and Maxent using the *dismo* R package version 1.3–5 (Hijmans et al. 2021) For each species, we generated 5000 pseudo‐absences by randomly sampling coordinates from the ecoregions where the species occurred and down‐weighted the absences in the model algorithms to balance the presence‐absence prevalence to 0.5 (Stockwell 1999). To assess model performance, we used a split‐sample approach (70% training data and 30% evaluation data) with 20 repetitions. Performance was measured using the area under the receiver operating characteristic (ROC) curve (AUC) (Bell and Fielding 1997; Hanley and McNeil 1982) and True Skill Statistics (TSS) values (Allouche et al. 2006). Binary classifications were done using the sensitivity‐specificity sum maximization approach in the R package *presenceAbsence* 1.1.9 (Freeman and Moisen 2008).

### 2.5 Mapping Vulnerability to Scorpion Sting

We mapped vulnerability to scorpion sting across the country considering two factors: (a) distance to cities which can provide primary health care services (accessibility to health care) and (b) exposure to medically important scorpions (binary habitat suitability maps). We calculated a distance‐to‐city layer as the distance of each grid cell to the nearest city using the *distance* function of the *raster* package 3.4–13 (Hijmans 2021). For each species, we masked the binary ecological niche model with the distance‐to‐city layer to get a vulnerability to scorpion sting map for each medically important scorpion. For the final risk maps, we multiplied the species ecological niche with the distance‐to‐city layer (Team 2021). From this, we extracted the values from the vulnerability to scorpion sting maps for each cell to get the vulnerability index.

## 3 Results

**FIGURE 2:**
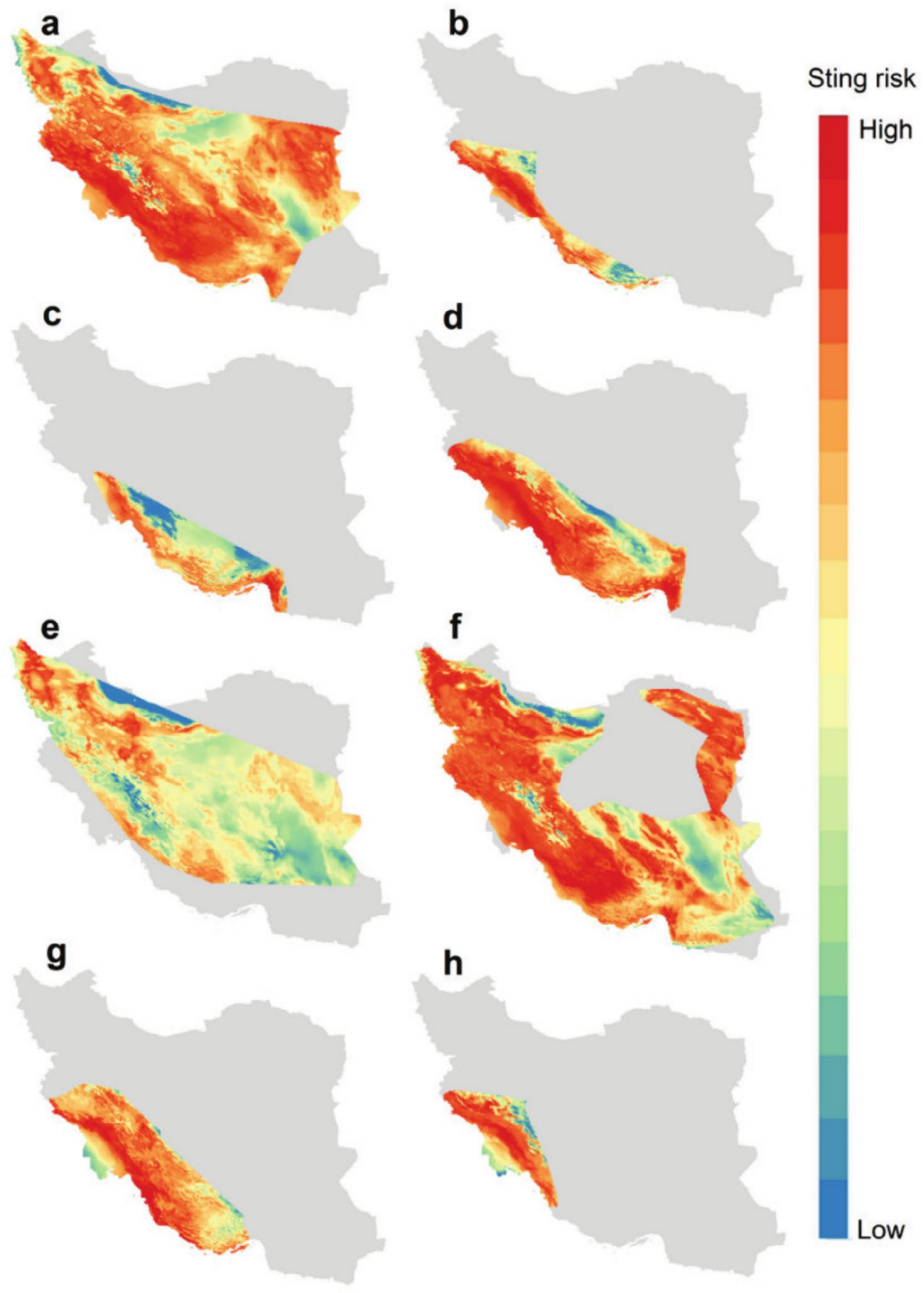
Scorpion sting risk based on the ensemble models of *Androctonus crassicauda* (a), *Buthacus macrocentrus* (b), *Hemiscorpius acanthocercus* (c), *Hemiscorpius lepturus* (d), *Mesobuthus caucasicus* (e), *Mesobuthus eupeus* (f), *Mesobuthus phillipsii* (g) and *Orthochirus iranus* (h).

**FIGURE 3:**
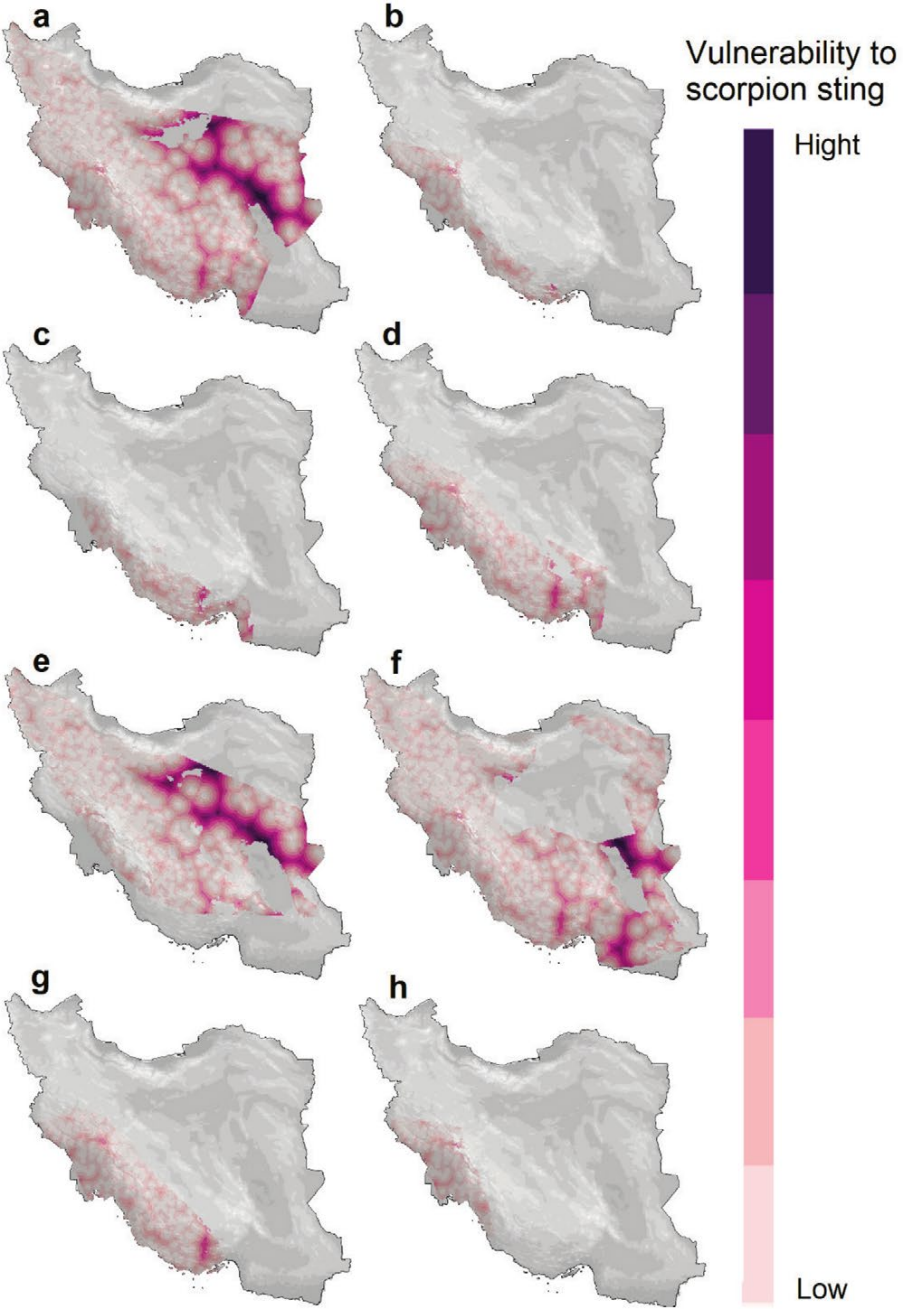
Vulnerability to scorpion sting maps of *Androctonus crassicauda* (a), *Buthacus macrocentrus* (b), *Hemiscorpius acanthocercus* (c), *Hemiscorpius lepturus* (d), *Mesobuthus caucasicus* (e), *Mesobuthus eupeus* (f), *Mesobuthus phillipsii* (g) and *Orthochirus iranus* (h). The vulnerability maps were created by considering the product of exposure to scorpions and distance to healthcare centers.

**FIGURE 4:**
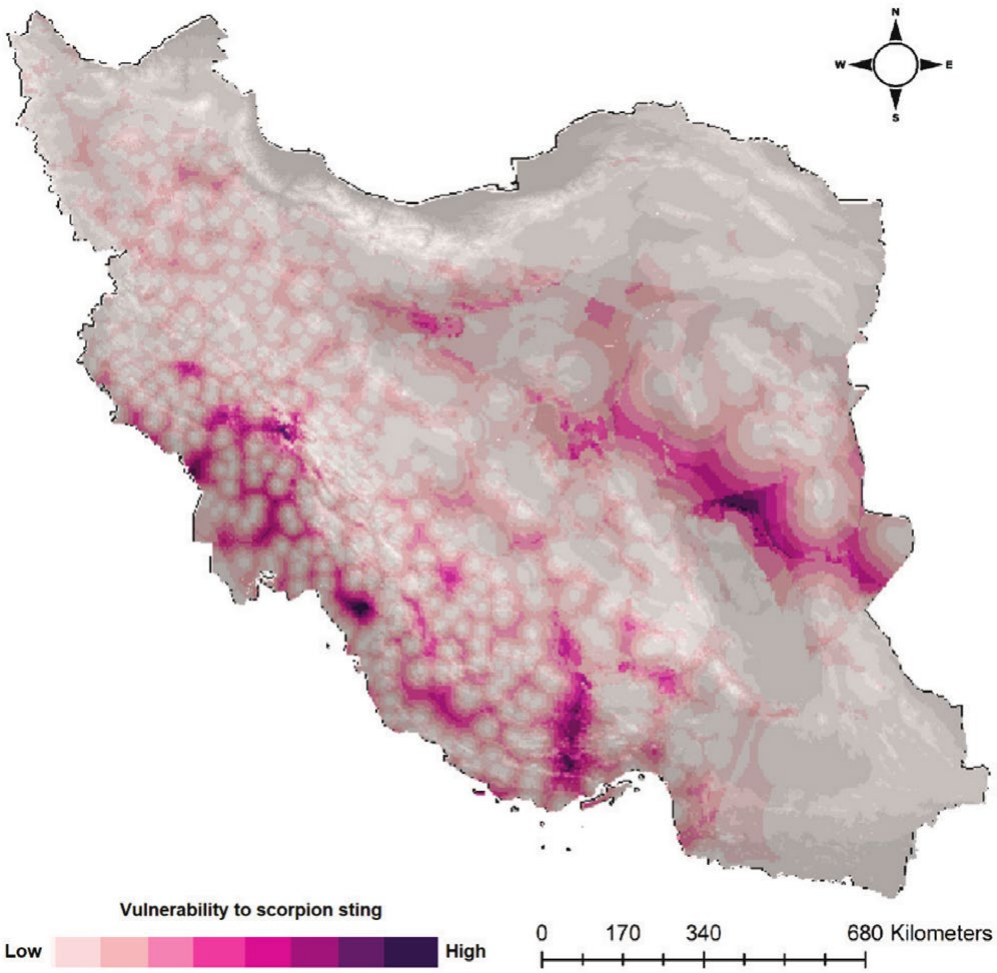
Vulnerability to scorpion sting map in Iran, which was created by considering the product of exposure to scorpions (all eight medically important scorpions) and distance to healthcare centers.

We identified sting risk areas for each species across their distribution ranges (Figure 2). *Androctonus crassicauda* has the widest distribution range. On the contrary, *Orthochirus iranus* has the smallest distribution range. Vulnerability to scorpion stings was modeled for each species separately (Figure 3) and in combination of all eight species (Figure 4) by considering the product of exposure to scorpions and distance to healthcare centers. Results showed that central parts of Iran and south and southwest of the country have the largest areas which are vulnerable to scorpion stings (Figure 4). Northeast, north, and northwest of the country have the lowest vulnerability to scorpion sting.

The calibrated ENMs performed well (Table 2). According to AUC values, *Hemiscorpius lepturus*' model has had the highest performance compared to the other seven species. While according to TSS values, *Orthochirus iranus'* outperformed other species.

**TABLE 2:** Results of model performance for the eight scorpions based on the AUC and TSS metrics.

Species	AUC	TSS
*Androctonus crassicauda*	0.911	0.824
*Buthacus macrocentrus*	0.805	0.791
*Mesobuthus caucasicus*	0.816	0.765
*Mesobuthus eupeus*	0.935	0.841
*Mesobuthus phillipsii*	0.874	0.725
*Orthochirus iranus*	0.965	0.851
*Hemiscorpius acanthocercus*	0.924	0.836
*Hemiscorpius lepturus*	0.977	0.849

## 4 Discussion

To facilitate scorpion sting management in Iran we combined medically important scorpion occurrence records from different sources to model their distribution and then identify high risk areas and the most vulnerable human populations across Iran. We found that most of the medically important scorpions are distributed in south west and southern parts of Iran making these regions high risk areas in the country. These regions showed suitable habitat for most of the venomous scorpions but are at the same time located in rural area, thus the access to medical treatment is difficult. Identifying these regions is critical to know where antivenin is most needed (Potet et al. 2021). This allows proper scorpion sting management by distributing antivenom among the vulnerable populations to reduce negative impacts of scorpion sting (Kafash et al. 2023). Our vulnerability scorpion sting map can guide antivenom distribution by the Ministry of Health and Medical Education across the country to make sure vulnerable population have access to antivenom. Travelers, tourists, farmers and shepherds need to take the necessary measures while in these areas, particularly to take antivenom with themselves when traveling to those regions.

Previous studies have shown that climate change will reshape species distributions, including those of scorpions (Rafinejad et al. 2020; Ureta et al. 2020). This was also shown for scorpion species in Iran (Kafash et al. 2023; Rafinejad et al. 2020). Thus, it is predicted that under climate change SSR and vulnerability to SSR will change as medically important scorpions' distributions are expected to change. Since scorpions are thermophilous, it is predicted that their distributions will increase under a warming climate (Rafinejad et al. 2020). We also expect that, while scorpions' ranges are expanding, they might shift their distribution within the human settlements as this was shown for *Mesobuthus phillipsii* (Kafash et al. 2023). This means that with climate change reshaping species distributions, it is paramount to consider the effects on SSR patterns (Kafash et al. 2023).

Modeling spatial patterns of SSR may be one strategy to reduce the incidence and improve treatment outcomes of scorpion sting. But one other particular and effective strategy to reduce and prevent scorpion stings is education (Daneshi et al. 2022; Kafash et al. 2023). It is certainly helpful to raise awareness about avoiding scorpion habitats and using protective clothing (Daneshi et al. 2022). These are simple solutions but still many at‐risk communities are being stung for not avoiding scorpion habitats and not wearing proper clothes. Here we identified high scorpion sting risk areas in Iran which can be the subject of awareness raising programs across the country. Considering that most of the victims seek traditional treatment, it is important to encourage them to seek health care instead (Dehghani and Arani 2015).

The magnitude of the impact of scorpion stings on human populations globally is surprisingly poorly understood, despite the ubiquity of the issue across much of Northern Africa and the Middle East, along with varying degrees of relevance for other regions around the World. Due to limited research on the topic, data on scorpion sting numbers are rarely collected, which in turn leads to a lack of evidence for the severity of the issue. Improving our understanding of the distribution of scorpions and SSR is a crucial first step to identify vulnerable communities, advise decision makers on where to provide improved medical resources, and to eventually collect data on the number of people impacted by scorpion stings.

In this study, we developed high resolution maps of SSR and SSR vulnerability in Iran. We showed that central parts of Iran and south and southwest of the country have the largest areas that are vulnerable to scorpion stings. It is important to note that research into more accessible, affordable antivenom and improved medical facilities can alleviate the health burden. As suggested for medically important venomous snakes (Pintor et al. 2021; Potet et al. 2021; Ruiz de Castañeda et al. 2022), collaborative efforts involving healthcare professionals, researchers, and communities are imperative in developing sustainable solutions, ultimately reducing the incidence and impact of scorpion stings on public health. Results of this study are useful for health authorities and decision‐makers to prioritize their measures and management programs to reduce and control scorpion stings in high‐risk areas.

Scorpion stings represent a medical challenge that is not limited to Iran but extends to other Middle Eastern and North African countries (Amr et al. 2021). Thus, we encourage this method to be applied in other countries to identify high‐priority areas for communities' awareness raising programs and antivenom distribution for the people most in need.

It should be noted that not all eight scorpion species studied hare are known with high medical concern and some like *Mesobuthus phillipsii* are known with low medical concern (Kafash et al. 2023) but considering that these species are not well studied in respect to their medical importance, symptoms, epidemiology and etc. we included them in this study because we believe high resolution SSR and SSR vulnerability maps can greatly facilitate these species management. We believe that further ecological and natural history studies are essential to better understand their behavior and habitat in Iran and set species specific management plan for scorpions of medical importance in the country.

## Author Contributions

**Anooshe Kafash:** conceptualization (lead), formal analysis (equal), investigation (lead), methodology (lead), writing – original draft (lead), writing – review and editing (lead). **Ahmad Ali Hanafi Bojd:** writing – review and editing (equal). **Anna Pintor:** writing – review and editing (equal). **Marc Grünig:** formal analysis (equal), writing – review and editing (equal). **Masoud Yousefi:** writing – review and editing (equal). **Gholamreza Hassanpour:** writing – review and editing (equal).

## conflicts of Interest

The authors declare no conflicts of interest.

## Supporting information


Table S1.

## Data Availability

The datasets generated and analyzed during the current study are available from the sources described in the manuscript and Supporting Information.
