# Supplementary material for: Applying Ensemble Ecological Niche Modeling to Identify High Risk Areas for Scorpions' Sting
Source: Ecol Evol. 2025 Jul 4;15(7):e71713. doi: 10.1002/ece3.71713 (PMC12231216; doi:10.1002/ece3.71713)
Supplement: Supplementary file 1 — Table S1. [file ECE3-15-e71713-s001.pdf]

Supplementary table S1. Distribution data of scorpions.

x	y	Species	x	Species	y
44.98333	38.56667	Buthacus macrocentrus	50.44889	Androctonus crassicauda	29.64222
45.01361	39.32639	Buthacus macrocentrus	51.08111	Androctonus crassicauda	28.71639
45.10917	39.30778	Buthacus macrocentrus	52.07889	Androctonus crassicauda	27.82639
45.11667	37.51667	Buthacus macrocentrus	51.5975	Androctonus crassicauda	27.89639
45.38333	36.93333	Buthacus macrocentrus	51.32583	Androctonus crassicauda	29.22944
45.6	38.91667	Buthacus macrocentrus	50.20028	Androctonus crassicauda	30.22833
45.91667	37.76667	Buthacus macrocentrus	49.74972	Androctonus crassicauda	30.71444
45.9666	33.75	Buthacus macrocentrus	54.77539	Androctonus crassicauda	26.76592
46	37.33333	Buthacus macrocentrus	54.52897	Androctonus crassicauda	26.75414
46.0147	33.0166	Buthacus macrocentrus	55.23311	Androctonus crassicauda	26.77683
46.11667	33.81667	Buthacus macrocentrus	55.36858	Androctonus crassicauda	26.84478
46.13333	33.8	Buthacus macrocentrus	56.05	Androctonus crassicauda	25.9
46.15	33.75	Buthacus macrocentrus	55.06667	Androctonus crassicauda	26.66667
46.15	33.76667	Buthacus macrocentrus	47.731	Androctonus crassicauda	32.42067
46.16667	33.73333	Buthacus macrocentrus	47.61537	Androctonus crassicauda	32.40175
46.1833	33.9333	Buthacus macrocentrus	47.68333	Androctonus crassicauda	32.73333
46.18333	38.08333	Buthacus macrocentrus	47.01667	Androctonus crassicauda	32.76667
46.18333	33.76667	Buthacus macrocentrus	49.52972	Androctonus crassicauda	30.63028
46.18333	36.5	Buthacus macrocentrus	48.48833	Androctonus crassicauda	31.46583
46.21667	37.41667	Buthacus macrocentrus	49.08333	Androctonus crassicauda	31.82611
46.2333	33.2666	Buthacus macrocentrus	49.56667	Androctonus crassicauda	30.93667
46.33333	35.03333	Buthacus macrocentrus	48.51778	Androctonus crassicauda	32.01528
46.53333	33.73333	Buthacus macrocentrus	49.23528	Androctonus crassicauda	31.27139
46.6	33.4	Buthacus macrocentrus	49.19472	Androctonus crassicauda	31.19833
46.6333	33.6333	Buthacus macrocentrus	48.26083	Androctonus crassicauda	32.18194

46.6666	33.75	Buthacus macrocentrus	48.96667	Androctonus crassicauda	31.5
47.0027	32.0125	Buthacus macrocentrus	49.71667	Androctonus crassicauda	33.38333
47.0075	32.4166	Buthacus macrocentrus	45.9666	Androctonus crassicauda	33.75
47.03333	35.31667	Buthacus macrocentrus	46.0147	Androctonus crassicauda	33.0166
47.05	33.3	Buthacus macrocentrus	46.1833	Androctonus crassicauda	33.9333
47.15306	33.69583	Buthacus macrocentrus	46.2333	Androctonus crassicauda	33.2666
47.2166	33.7333	Buthacus macrocentrus	46.5333	Androctonus crassicauda	33.7333
47.3666	33.15	Buthacus macrocentrus	46.6	Androctonus crassicauda	33.4
47.43333	34.38333	Buthacus macrocentrus	46.6333	Androctonus crassicauda	33.6333
47.61537	32.40175	Buthacus macrocentrus	46.6666	Androctonus crassicauda	33.75
47.62467	32.41267	Buthacus macrocentrus	47.0027	Androctonus crassicauda	32.0125
47.663	32.772	Buthacus macrocentrus	47.0075	Androctonus crassicauda	32.4166
47.7	37.45	Buthacus macrocentrus	47.05	Androctonus crassicauda	33.3
47.71667	33.13333	Buthacus macrocentrus	47.2166	Androctonus crassicauda	33.7333
47.731	32.42067	Buthacus macrocentrus	47.3666	Androctonus crassicauda	33.15
48.1625	36.835	Buthacus macrocentrus	48.4019	Androctonus crassicauda	32.3824
48.23333	32.06667	Buthacus macrocentrus	49.6	Androctonus crassicauda	31.2833
48.25722	32.77083	Buthacus macrocentrus	48.0833	Androctonus crassicauda	31.826
48.31861	31.85444	Species	x	Androctonus crassicauda	y
48.38333	36.66667	Mesobuthus caucasicus	48.55	Androctonus crassicauda	37.58333
48.38389	36.69389	Mesobuthus caucasicus	46.18333	Androctonus crassicauda	38.08333
48.4019	32.3824	Mesobuthus caucasicus	46	Androctonus crassicauda	37.33333
48.43333	34.75	Mesobuthus caucasicus	45.6	Androctonus crassicauda	38.91667
48.45	34.76667	Mesobuthus caucasicus	45.71667	Androctonus crassicauda	38.41667
48.48333	34.75	Mesobuthus caucasicus	51.43333	Androctonus crassicauda	34.03333
48.55	37.58333	Mesobuthus caucasicus	51.71667	Androctonus crassicauda	33.6
48.65	34.78333	Mesobuthus caucasicus	54.4	Androctonus crassicauda	28.36667

48.79611	36.45722	Mesobuthus caucasicus	51.86833	Androctonus crassicauda	29.31792
48.86667	33.2	Mesobuthus caucasicus	47.15	Androctonus crassicauda	34.3
48.88333	31.23333	Mesobuthus caucasicus	50.18333	Androctonus crassicauda	30.58333
48.96667	31.5	Mesobuthus caucasicus	50.78333	Androctonus crassicauda	30.33333
48.99083	33.5475	Mesobuthus caucasicus	49.71667	Androctonus crassicauda	34.11667
49.24056	31.23194	Mesobuthus caucasicus	50.95	Androctonus crassicauda	34.63333
49.30683	34.022	Mesobuthus caucasicus	53.43333	Androctonus crassicauda	35.56667
49.36667	31.25	Mesobuthus caucasicus	61.5	Androctonus crassicauda	31
49.37083	31.92139	Mesobuthus caucasicus	61.21667	Androctonus crassicauda	28.23333
49.38333	36.76667	Mesobuthus caucasicus	60.1	Androctonus crassicauda	33.03333
49.4058	30.891	Mesobuthus caucasicus	51.55	Androctonus crassicauda	35.56667
49.6	31.2833	Mesobuthus caucasicus	45.11667	Androctonus crassicauda	37.51667
49.65	36.05	Mesobuthus caucasicus	47.1	Androctonus crassicauda	36.38333
49.71667	33.38333	Mesobuthus caucasicus	46.08333	Androctonus crassicauda	36.96667
49.95	36.25	Mesobuthus caucasicus	44.75	Androctonus crassicauda	38.2
50.41417	30.5225	Mesobuthus caucasicus	45.07667	Androctonus crassicauda	39.32778
50.4488	29.6222	Mesobuthus caucasicus	45.04833	Androctonus crassicauda	39.29556
50.44889	29.64222	Mesobuthus caucasicus	45.04167	Androctonus crassicauda	39.37389
50.46786	33.86906	Mesobuthus caucasicus	45.10083	Androctonus crassicauda	39.30778
50.65331	36.05961	Mesobuthus caucasicus	44.99	Androctonus crassicauda	38.91
50.74306	30.73972	Mesobuthus caucasicus	44.6167	Androctonus crassicauda	39.28
50.78489	35.95714	Mesobuthus caucasicus	54.41667	Androctonus crassicauda	31.86667
50.8293	31.5102	Species	x	Androctonus crassicauda	y
50.83279	32.36204	Mesobuthus eupeus	50.97278	Androctonus crassicauda	35.91019
50.95	34.63333	Mesobuthus eupeus	50.78489	Androctonus crassicauda	35.95714
50.9585	35.93136	Mesobuthus eupeus	50.93806	Androctonus crassicauda	35.94142
51.04263	31.56556	Mesobuthus eupeus	50.9585	Androctonus crassicauda	35.93136

51.04389	31.53389	Mesobuthus eupeus	51.00817	Androctonus crassicauda	35.933
51.04473	31.55325	Mesobuthus eupeus	51.03197	Androctonus crassicauda	35.98419
51.0675	31.53833	Mesobuthus eupeus	51.17311	Androctonus crassicauda	36.15467
51.08111	28.71639	Mesobuthus eupeus	50.66778	Androctonus crassicauda	36.12272
51.15	32.66667	Mesobuthus eupeus	50.67944	Androctonus crassicauda	36.15114
51.3166	34.2833	Mesobuthus eupeus	50.885	Androctonus crassicauda	36.2025
51.34111	29.56222	Mesobuthus eupeus	51.14325	Androctonus crassicauda	36.16031
51.4166	34.0833	Mesobuthus eupeus	48.55	Androctonus crassicauda	37.58333
51.43333	34.03333	Mesobuthus eupeus	50.41667	Androctonus crassicauda	29.66667
51.5975	27.89639	Mesobuthus eupeus	48.51667	Androctonus crassicauda	32
51.60917	29.65306	Mesobuthus eupeus	50.88333	Androctonus crassicauda	32.35
51.83167	29.48611	Mesobuthus eupeus	51.25	Androctonus crassicauda	31.96667
51.86833	29.31792	Mesobuthus eupeus	50.65	Androctonus crassicauda	32
51.87333	29.59111	Mesobuthus eupeus	45.91667	Androctonus crassicauda	37.76667
52.17472	30.29972	Mesobuthus eupeus	46.03333	Androctonus crassicauda	37.95
52.24194	29.4175	Mesobuthus eupeus	47.08333	Androctonus crassicauda	38.48333
52.58333	29.61667	Mesobuthus eupeus	47.03333	Androctonus crassicauda	37.48333
52.82194	35.71194	Mesobuthus eupeus	45.6	Androctonus crassicauda	38.91667
53.01667	28.3	Mesobuthus eupeus	45.71667	Androctonus crassicauda	38.41667
53.11139	28.24278	Mesobuthus eupeus	47.7	Androctonus crassicauda	37.45
53.23333	31.11667	Mesobuthus eupeus	51.48333	Androctonus crassicauda	32.03333
53.43333	35.56667	Mesobuthus eupeus	51.43333	Androctonus crassicauda	34.03333
53.96667	32.23333	Mesobuthus eupeus	51.15	Androctonus crassicauda	32.66667
54.05417	28.24583	Mesobuthus eupeus	51.16667	Androctonus crassicauda	33.98333
54.21667	31.73333	Mesobuthus eupeus	52.91667	Androctonus crassicauda	30.08333
54.32583	27.70833	Mesobuthus eupeus	51.4	Androctonus crassicauda	29.5
54.36667	27.2	Mesobuthus eupeus	51.63333	Androctonus crassicauda	29.55

54.38333	30.05	Mesobuthus eupeus	52.6	Androctonus crassicauda	28.76667
54.4	31.58333	Mesobuthus eupeus	53.06667	Androctonus crassicauda	28.41667
54.41667	31.55	Mesobuthus eupeus	55.43333	Androctonus crassicauda	28.16667
54.41667	31.86667	Mesobuthus eupeus	52.51667	Androctonus crassicauda	28.91667
54.58333	26.93333	Mesobuthus eupeus	52.3725	Androctonus crassicauda	29.00778
54.89628	26.75139	Mesobuthus eupeus	52.91778	Androctonus crassicauda	29.43028
55.06667	26.66667	Mesobuthus eupeus	52.82806	Androctonus crassicauda	29.79222
55.09472	30.02861	Mesobuthus eupeus	52.33056	Androctonus crassicauda	31.13417
55.41667	31.58333	Mesobuthus eupeus	52.2	Androctonus crassicauda	31.73333
55.53333	31.68333	Mesobuthus eupeus	52.17472	Androctonus crassicauda	30.29972
55.58333	26.93333	Mesobuthus eupeus	53.05806	Androctonus crassicauda	28.41667
55.72222	29.51139	Mesobuthus eupeus	53.10778	Androctonus crassicauda	28.24417
55.82153	27.135	Mesobuthus eupeus	53.13944	Androctonus crassicauda	29.13194
55.89833	30.04806	Mesobuthus eupeus	53.21083	Androctonus crassicauda	29.10083
56	26.8333	Mesobuthus eupeus	53.08444	Androctonus crassicauda	28.25444
56.01667	31.86667	Mesobuthus eupeus	53.05111	Androctonus crassicauda	29.40583
56.07833	30.53944	Mesobuthus eupeus	52.89667	Androctonus crassicauda	28.50556
56.09944	30.08	Mesobuthus eupeus	52.79833	Androctonus crassicauda	28.54333
56.11667	26.83333	Mesobuthus eupeus	52.62528	Androctonus crassicauda	28.45778
56.16667	26.9	Mesobuthus eupeus	52.67028	Androctonus crassicauda	28.46417
56.25	26.25	Mesobuthus eupeus	52.86722	Androctonus crassicauda	29.51861
56.33333	27.26667	Mesobuthus eupeus	54.05417	Androctonus crassicauda	28.24583
56.4	27.4	Mesobuthus eupeus	54.10444	Androctonus crassicauda	29.09417
56.58861	30.795	Mesobuthus eupeus	54.4	Androctonus crassicauda	28.36667
56.92167	30.23444	Mesobuthus eupeus	51.60917	Androctonus crassicauda	29.65306
57.06667	27.13333	Mesobuthus eupeus	51.87333	Androctonus crassicauda	29.59111
57.16611	30.15583	Mesobuthus eupeus	51.34111	Androctonus crassicauda	29.56222

57.19056	30.12361	Mesobuthus eupeus	51.86833	Androctonus crassicauda	29.31792
57.26861	30.02556	Mesobuthus eupeus	55.83333	Androctonus crassicauda	37.38333
57.29922	26.08036	Mesobuthus eupeus	56	Androctonus crassicauda	37.33333
57.63333	26.45	Mesobuthus eupeus	49.4	Androctonus crassicauda	36.78333
57.93667	27.63722	Mesobuthus eupeus	48.45	Androctonus crassicauda	35.4
58.33333	29.11667	Mesobuthus eupeus	48.75	Androctonus crassicauda	30.66667
58.65	34.36667	Mesobuthus eupeus	48.65	Androctonus crassicauda	34.78333
58.83333	28.98333	Mesobuthus eupeus	48.43333	Androctonus crassicauda	34.75
60	33.56667	Mesobuthus eupeus	48.41667	Androctonus crassicauda	34.81667
60.1	33.03333	Mesobuthus eupeus	48.48333	Androctonus crassicauda	34.75
60.15	34.53333	Mesobuthus eupeus	48.45	Androctonus crassicauda	34.76667
60.88333	29.95	Mesobuthus eupeus	55.86667	Androctonus crassicauda	26.63333
61.48333	30.95	Mesobuthus eupeus	55.58333	Androctonus crassicauda	26.93333
61.51667	31.01667	Mesobuthus eupeus	57.06667	Androctonus crassicauda	27.13333
61.61667	31.11667	Mesobuthus eupeus	54.36667	Androctonus crassicauda	27.2
x	y	Mesobuthus eupeus	56.11667	Species	26.83333
55.0685	26.6777	Mesobuthus eupeus	57.16281	Mesobuthus phillipsii	26.99328
55.06872222	26.67783333	Mesobuthus eupeus	57.2575	Mesobuthus phillipsii	26.85081
54.8837	26.7432	Mesobuthus eupeus	57.3205	Mesobuthus phillipsii	26.86592
54.88380556	26.74633333	Mesobuthus eupeus	56.94219	Mesobuthus phillipsii	27.40222
54.8962	26.7513	Mesobuthus eupeus	56.99508	Mesobuthus phillipsii	27.4175
54.89627778	26.75138889	Mesobuthus eupeus	57.29922	Mesobuthus phillipsii	26.08036
54.5288	26.7541	Mesobuthus eupeus	57.83739	Mesobuthus phillipsii	25.8365
54.52897222	26.75413889	Mesobuthus eupeus	57.84464	Mesobuthus phillipsii	25.84808
54.7753	26.7659	Mesobuthus eupeus	57.82842	Mesobuthus phillipsii	25.92178
54.77538889	26.76591667	Mesobuthus eupeus	57.4965	Mesobuthus phillipsii	25.87636
55.233	26.7767	Mesobuthus eupeus	57.09528	Mesobuthus phillipsii	26.62519

55.23311111	26.77683333	Mesobuthus eupeus	56.8355	Mesobuthus phillipsii	27.38075
55.4983	27.0779	Mesobuthus eupeus	55.66786	Mesobuthus phillipsii	27.20039
55.49841667	27.07805556	Mesobuthus eupeus	56.18578	Mesobuthus phillipsii	27.16544
53.4395	27.0855	Mesobuthus eupeus	55.82153	Mesobuthus phillipsii	27.135
53.43958333	27.08561111	Mesobuthus eupeus	56.4	Mesobuthus phillipsii	27.4
53.4905	27.0876	Mesobuthus eupeus	56.16667	Mesobuthus phillipsii	26.9
53.49061111	27.08769444	Mesobuthus eupeus	55.06667	Mesobuthus phillipsii	26.66667
53.3897	27.0978	Mesobuthus eupeus	46.4	Mesobuthus phillipsii	33.6
53.38977778	27.09786111	Mesobuthus eupeus	46.28333	Mesobuthus phillipsii	33.83333
54.2318	27.5111	Mesobuthus eupeus	46.13333	Mesobuthus phillipsii	33.8
54.23194444	27.51111111	Mesobuthus eupeus	47.68333	Mesobuthus phillipsii	32.73333
54.4527	27.5229	Mesobuthus eupeus	46.18333	Mesobuthus phillipsii	33.93333
54.45277778	27.52305556	Mesobuthus eupeus	47.01667	Mesobuthus phillipsii	33.31667
52.8433	27.5741	Mesobuthus eupeus	46.45	Mesobuthus phillipsii	33.65
52.84333333	27.57416667	Mesobuthus eupeus	46.26667	Mesobuthus phillipsii	33.41667
52.703	27.598	Mesobuthus eupeus	46.53333	Mesobuthus phillipsii	33.73333
52.70305556	27.59805556	Mesobuthus eupeus	46.83333	Mesobuthus phillipsii	33.16667
54.3266	27.6682	Mesobuthus eupeus	57.71792	Mesobuthus phillipsii	28.60639
54.32666667	27.66833333	Mesobuthus eupeus	57.92565	Mesobuthus phillipsii	29.0895
52.608	27.7041	Mesobuthus eupeus	57.9	Mesobuthus phillipsii	28.96667
52.60805556	27.70416667	Mesobuthus eupeus	56.82694	Mesobuthus phillipsii	28.44028
52.07888889	27.82638889	Mesobuthus eupeus	57.78361	Mesobuthus phillipsii	28.50083
55.71666667	28.16666667	Mesobuthus eupeus	57.79694	Mesobuthus phillipsii	28.51694
51.12777778	28.47	Mesobuthus eupeus	57.76111	Mesobuthus phillipsii	28.61139
51.36361111	28.69611111	Mesobuthus eupeus	57.93944	Mesobuthus phillipsii	27.64389
51.34722222	28.8625	Mesobuthus eupeus	55.89833	Mesobuthus phillipsii	30.04806
51.31194444	28.88138889	Mesobuthus eupeus	55.09472	Mesobuthus phillipsii	30.02861
52.51666667	28.91666667	Mesobuthus eupeus	55.97833	Mesobuthus phillipsii	29.40028

52.0158	29.1754	Mesobuthus eupeus	56.6805<br>6	Mesobuthus phillipsii	29.24194
x	y	Mesobuthus eupeus	56.8388<br>9	Species	29.34111
50.41666<br>667	29.66666<br>667	Mesobuthus eupeus	56.6144<br>4	Orthochirus iranus	29.11556
50.44888<br>889	29.64222<br>222	Mesobuthus eupeus	56.6152<br>8	Orthochirus iranus	29.13861
51.08111<br>111	28.71638<br>889	Mesobuthus eupeus	56.6205<br>6	Orthochirus iranus	29.15583
51.36361<br>111	28.69611<br>111	Mesobuthus eupeus	56.4866<br>7	Orthochirus iranus	29.25639
50.20027<br>778	30.31333<br>333	Mesobuthus eupeus	56.8355<br>6	Orthochirus iranus	31.36222
47.731	32.42066<br>667	Mesobuthus eupeus	56.58	Orthochirus iranus	29.95
47.61536<br>667	32.40175	Mesobuthus eupeus	46.2333<br>3	Orthochirus iranus	34.26667
46.18333<br>333	33.93333<br>333	Mesobuthus eupeus	48.8833<br>3	Orthochirus iranus	31.23333
47.01666<br>667	32.76666<br>667	Mesobuthus eupeus	49.6166<br>7	Orthochirus iranus	30.75
46.45	33.65	Mesobuthus eupeus	48.3166<br>7	Orthochirus iranus	32.46667
46.26666<br>667	33.41666<br>667	Mesobuthus eupeus	48.4333<br>3	Orthochirus iranus	32.43333
46.53333<br>333	33.73333<br>333	Mesobuthus eupeus	48.5	Orthochirus iranus	32.78333
48.51666<br>667	32	Mesobuthus eupeus	48.3166<br>7	Orthochirus iranus	32.76667
49.96027<br>778	31.45666<br>667	Mesobuthus eupeus	51.6	Orthochirus iranus	30.65
49.52972<br>222	30.63027<br>778	Mesobuthus eupeus	50.9	Orthochirus iranus	30.93333
49.08333<br>333	31.82611<br>111	Mesobuthus eupeus	47.0333<br>3	Orthochirus iranus	35.31667
50.74555<br>556	30.56222<br>222	Mesobuthus eupeus	49.7166<br>7	Orthochirus iranus	33.38333
47.71083<br>333	33.08944<br>444	Mesobuthus eupeus	48.8666<br>7	Orthochirus iranus	33.2
47.09166<br>667	33.505	Mesobuthus eupeus	49.3003<br>3	Orthochirus iranus	34.03731
49.71666<br>667	33.38333<br>333	Mesobuthus eupeus	49.3068<br>3	Orthochirus iranus	34.022
50.4166	29.7333	Mesobuthus eupeus	50.3300<br>8	Orthochirus iranus	33.80894
45.9666	33.75	Mesobuthus eupeus	50.2405	Orthochirus iranus	33.73497
46.0147	33.0166	Mesobuthus eupeus	49.8681<br>4	Orthochirus iranus	33.97994
46.2333	33.2666	Mesobuthus eupeus	49.9917<br>8	Orthochirus iranus	33.83697
46.6	33.4	Mesobuthus eupeus	50.1621<br>1	Orthochirus iranus	33.78344

46.6333	33.6333	Mesobuthus eupeus	50.06186	Orthochirus iranus	33.81589
46.6666	33.75	Mesobuthus eupeus	49.52111	Orthochirus iranus	34.72444
47.0027	32.0125	Mesobuthus eupeus	49.6675	Orthochirus iranus	34.43306
47.0075	32.4166	Mesobuthus eupeus	50.19111	Orthochirus iranus	34.28444
47.05	33.3	Mesobuthus eupeus	50.14611	Orthochirus iranus	34.25389
47.2166	33.7333	Mesobuthus eupeus	50.06083	Orthochirus iranus	34.20389
47.3666	33.15	Mesobuthus eupeus	49.61889	Orthochirus iranus	33.94972
49.4058	30.891	Mesobuthus eupeus	49.61583	Orthochirus iranus	33.98278
x	y	Mesobuthus eupeus	51.4	Species	36.61667
48.55	38.43333	Mesobuthus eupeus	51.3	Hemiscorpius lepturus	36.23333
51.83222	27.99556	Mesobuthus eupeus	53.1	Hemiscorpius lepturus	36.56667
51.34722	28.865	Mesobuthus eupeus	51.2166	Hemiscorpius lepturus	36.5166
51.26667	29.39083	Mesobuthus eupeus	53.54	Hemiscorpius lepturus	36.23
50.20028	30.22833	Mesobuthus eupeus	51.28333	Hemiscorpius lepturus	36.43333
49.60056	32.49111	Mesobuthus eupeus	57.3	Hemiscorpius lepturus	37.43333
51.0675	31.53833	Mesobuthus eupeus	49.95	Hemiscorpius lepturus	36.25
50.77167	31.77972	Mesobuthus eupeus	50.56667	Hemiscorpius lepturus	36.43333
50.82882	31.75549	Mesobuthus eupeus	50.86667	Hemiscorpius lepturus	34.38333
52.88333	29.93333	Mesobuthus eupeus	60.85	Hemiscorpius lepturus	34.68333
52.58333	29.61667	Mesobuthus eupeus	60.8	Hemiscorpius lepturus	34.8
52.91667	30.08333	Mesobuthus eupeus	59.33	Hemiscorpius lepturus	36.033
52.68333	31.23333	Mesobuthus eupeus	59.37	Hemiscorpius lepturus	36.116
51.5	30.38333	Mesobuthus eupeus	58.754	Hemiscorpius lepturus	34.185
52.93056	29.97222	Mesobuthus eupeus	60.583	Hemiscorpius lepturus	36.316
51.92806	29.96639	Mesobuthus eupeus	60.03333	Hemiscorpius lepturus	34.58333
54.16667	27.56667	Mesobuthus eupeus	59.36667	Hemiscorpius lepturus	35.28333
54.4	28.36667	Mesobuthus eupeus	60.4	Hemiscorpius lepturus	35.18333
51.60917	29.65306	Mesobuthus eupeus	58.73333	Hemiscorpius lepturus	34.41667
51.87333	29.59111	Mesobuthus eupeus	50.08267	Hemiscorpius lepturus	30.03186
50.25062	37.2487	Mesobuthus eupeus	49.07014	Hemiscorpius lepturus	30.48008
48.75	30.66667	Mesobuthus eupeus	53.43333	Hemiscorpius lepturus	35.56667
56.25	26.25	Mesobuthus eupeus	60.46	Hemiscorpius lepturus	27.17
54.58333	26.93333	Mesobuthus eupeus	60.77	Hemiscorpius lepturus	28.74

53.01667	28.3	Mesobuthus eupeus	61.48	Hemiscorpius lepturus	31.04
55.58333	26.93333	Mesobuthus eupeus	61.33333	Hemiscorpius lepturus	30.85
57.06667	27.13333	Mesobuthus eupeus	61.35	Hemiscorpius lepturus	30.75
54.36667	27.2	Mesobuthus eupeus	60.51667	Hemiscorpius lepturus	30.96667
56.11667	26.83333	Mesobuthus eupeus	60.5	Hemiscorpius lepturus	29.43333
57.63333	26.45	Mesobuthus eupeus	59.76667	Hemiscorpius lepturus	26.05
56.94219	27.40222	Mesobuthus eupeus	62.28333	Hemiscorpius lepturus	26.86667
56.99508	27.4175	Mesobuthus eupeus	61.43333	Hemiscorpius lepturus	30.85
47.663	32.772	Mesobuthus eupeus	59.5044	Hemiscorpius lepturus	31.821
47.421	32.86367	Mesobuthus eupeus	60.1	Hemiscorpius lepturus	33.03333
46.68867	32.92367	Mesobuthus eupeus	59.03333	Hemiscorpius lepturus	34.01667
47.9545	32.35767	Mesobuthus eupeus	51.55	Hemiscorpius lepturus	35.56667
46.18333	33.76667	Mesobuthus eupeus	52.81861	Hemiscorpius lepturus	35.68861
46.13333	33.8	Mesobuthus eupeus	52.57806	Hemiscorpius lepturus	35.6675
47.68333	32.73333	Mesobuthus eupeus	52.82194	Hemiscorpius lepturus	35.71194
48.50167	36.77778	Mesobuthus eupeus	52.86528	Mesobuthus eupeus	35.74028
48.27278	36.61694	Mesobuthus eupeus	52.84611	Mesobuthus eupeus	35.78444
48.79611	36.45722	Mesobuthus eupeus	52.9225	Mesobuthus eupeus	35.82528
48.38389	36.69389	Mesobuthus eupeus	52.65583	Mesobuthus eupeus	35.75194
48.67084	36.55452	Mesobuthus eupeus	52.73472	Mesobuthus eupeus	35.77556
48.20778	36.77611	Mesobuthus eupeus	52.72194	Mesobuthus eupeus	35.795
47.64167	36.58417	Mesobuthus eupeus	52.02028	Mesobuthus eupeus	35.7175
50.8293	31.5102	Mesobuthus eupeus	50.96806	Mesobuthus eupeus	35.48611
45.9666	33.75	Mesobuthus eupeus	51.71222	Mesobuthus eupeus	35.56528
46.1833	33.9333	Mesobuthus eupeus	51.42167	Mesobuthus eupeus	35.28806
46.5333	33.7333	Mesobuthus eupeus	51.67417	Mesobuthus eupeus	35.99278
46.6	33.4	Mesobuthus eupeus	51.68889	Mesobuthus eupeus	35.58528
46.6333	33.6333	Mesobuthus eupeus	52.0475	Mesobuthus eupeus	35.8575
46.6666	33.75	Mesobuthus eupeus	51.68806	Mesobuthus eupeus	35.73194
47.0027	32.0125	Mesobuthus eupeus	51.39167	Mesobuthus eupeus	35.05778

47.0077	32.0158	Mesobuthus eupeus	44.4	Mesobuthus eupeus	39.38333
47.05	33.3	Mesobuthus eupeus	45.03333	Mesobuthus eupeus	39.33333
47.2166	33.7333	Mesobuthus eupeus	45.11667	Mesobuthus eupeus	37.51667
47.3666	33.15	Mesobuthus eupeus	44.98333	Mesobuthus eupeus	38.56667
46.15	33.75	Mesobuthus eupeus	44.5	Mesobuthus eupeus	3.283333
46.15	33.7666	Mesobuthus eupeus	46.7	Mesobuthus eupeus	36.78333
46.1666	33.7333	Mesobuthus eupeus	44.75	Mesobuthus eupeus	38.2
46.1833	33.7666	Mesobuthus eupeus	44.93	Mesobuthus eupeus	37.46222
46.1333	33.8	Mesobuthus eupeus	44.91806	Mesobuthus eupeus	37.71361
48.50167	36.77778	Mesobuthus eupeus	44.92778	Mesobuthus eupeus	38.32222
48.27278	36.61694	Mesobuthus eupeus	44.79583	Mesobuthus eupeus	38.20056
48.79611	36.45722	Mesobuthus eupeus	44.78861	Mesobuthus eupeus	38.25556
48.38389	36.69389	Mesobuthus eupeus	44.12167	Mesobuthus eupeus	36.69444
48.67084	36.55452	Mesobuthus eupeus	45.36556	Mesobuthus eupeus	36.78222
48.20778	36.77611	Mesobuthus eupeus	45.39722	Mesobuthus eupeus	36.77
47.64167	36.58417	Mesobuthus eupeus	45.14722	Mesobuthus eupeus	36.71583
50.8293	31.5102	Mesobuthus eupeus	45.13917	Mesobuthus eupeus	36.98556
45.9666	33.75	Mesobuthus eupeus	45.11639	Mesobuthus eupeus	37.05472
46.1833	33.9333	Mesobuthus eupeus	44.4075	Mesobuthus eupeus	38.47917
46.5333	33.7333	Mesobuthus eupeus	44.95278	Mesobuthus eupeus	38.50444
46.6	33.4	Mesobuthus eupeus	45.65083	Mesobuthus eupeus	36.18889
46.6333	33.6333	Mesobuthus eupeus	45.33333	Mesobuthus eupeus	36.4
46.6666	33.75	Mesobuthus eupeus	45.4175	Mesobuthus eupeus	36.26583
47.0027	32.0125	Mesobuthus eupeus	44.37917	Mesobuthus eupeus	39.12333
47.0077	32.0158	Mesobuthus eupeus	44.4675	Mesobuthus eupeus	39.05306
47.05	33.3	Mesobuthus eupeus	44.40222	Mesobuthus eupeus	38.9975
47.2166	33.7333	Mesobuthus eupeus	44.38389	Mesobuthus eupeus	39.03722
47.3666	33.15	Mesobuthus eupeus	53.23333	Mesobuthus eupeus	31.11667
46.15	33.75	Mesobuthus eupeus	55.41667	Mesobuthus eupeus	31.58333
46.15	33.7666	Mesobuthus eupeus	54.38333	Mesobuthus eupeus	30.05

46.1666	33.7333	Mesobuthus eupeus	54.41667	Mesobuthus eupeus	31.55
48.96667	38.81667	Mesobuthus eupeus	53.96667	Mesobuthus eupeus	32.23333
48.48333	36.68333	Mesobuthus eupeus	54.21667	Mesobuthus eupeus	31.73333
47.66667	36.73333	Mesobuthus eupeus	54.41667	Mesobuthus eupeus	31.86667
48.2	36.76667	Mesobuthus eupeus	55.5	Mesobuthus eupeus	31.7
48.54417	36.72861	Mesobuthus eupeus	54.21667	Mesobuthus eupeus	31.73333
48.20444	36.61083	Mesobuthus eupeus	54.4	Mesobuthus eupeus	31.51667
48.36472	36.56639	Mesobuthus eupeus	48.96667	Mesobuthus eupeus	38.81667
